# Microfluidic Formulation of DNA-Loaded Multicomponent Lipid Nanoparticles for Gene Delivery

**DOI:** 10.3390/pharmaceutics13081292

**Published:** 2021-08-19

**Authors:** Erica Quagliarini, Serena Renzi, Luca Digiacomo, Francesca Giulimondi, Barbara Sartori, Heinz Amenitsch, Valentina Tassinari, Laura Masuelli, Roberto Bei, Lishan Cui, Junbiao Wang, Augusto Amici, Cristina Marchini, Daniela Pozzi, Giulio Caracciolo

**Affiliations:** 1 Department of Chemistry, “Sapienza” University of Rome, 00185 Rome, Italy; erica.quagliarini@uniroma1.it; 2 Department of Molecular Medicine, “Sapienza” University of Rome, 00161 Rome, Italy; serena.renzi@uniroma1.it (S.R.); luca.digiacomo@uniroma1.it (L.D.); francesca.giulimondi@uniroma1.it (F.G.); valentina.tassinari@uniroma1.it (V.T.); 3 Institute of inorganic Chemistry, Graz University of Technology, 8010 Graz, Austria; barbara.sartori@tugraz.at (B.S.); amenitsch@tugraz.at (H.A.); 4 Department of Experimental Medicine, “Sapienza” University of Rome, 00185 Rome, Italy; laura.masuelli@uniroma1.it; 5 Department of Clinical Sciences and Translational Medicine, University of Rome “Tor Vergata”, 00133 Rome, Italy; bei@med.uniroma2.it; 6 School of Biosciences and Veterinary Medicine, University of Camerino, 62032 Camerino, Italy; lishan.cui@unicam.it (L.C.); junbiao.wang@unicam.it (J.W.); augusto.amici@unicam.it (A.A.); cristina.marchini@unicam.it (C.M.)

**Keywords:** lipid nanoparticles, microfluidics, transfection efficiency, lipofectamine

## Abstract

In recent years, lipid nanoparticles (LNPs) have gained considerable attention in numerous research fields ranging from gene therapy to cancer immunotherapy and DNA vaccination. While some RNA-encapsulating LNP formulations passed clinical trials, DNA-loaded LNPs have been only marginally explored so far. To fulfil this gap, herein we investigated the effect of several factors influencing the microfluidic formulation and transfection behavior of DNA-loaded LNPs such as PEGylation, total flow rate (TFR), concentration and particle density at the cell surface. We show that PEGylation and post-synthesis sample concentration facilitated formulation of homogeneous and small size LNPs with high transfection efficiency and minor, if any, cytotoxicity on human Embryonic Kidney293 (HEK-293), spontaneously immortalized human keratinocytes (HaCaT), immortalized keratinocytes (N/TERT) generated from the transduction of human primary keratinocytes, and epidermoid cervical cancer (CaSki) cell lines. On the other side, increasing TFR had a detrimental effect both on the physicochemical properties and transfection properties of LNPs. Lastly, the effect of particle concentration at the cell surface on the transfection efficiency (TE) and cell viability was largely dependent on the cell line, suggesting that its case-by-case optimization would be necessary. Overall, we demonstrate that fine tuning formulation and microfluidic parameters is a vital step for the generation of highly efficient DNA-loaded LNPs.

## 1. Introduction

### 1.1. Lipid-Based Gene Delivery Systems

To exert their function properly, nucleic acids (NAs) such as messenger RNA (mRNA), short interfering RNA (siRNA), and plasmid DNA (pDNA) need to reach their target tissue without any alterations of their complex structures and, subsequently, interact with cytosol and/or nucleus of target cells [1]. Nevertheless, free NAs are highly susceptible to rapid degradation in biological media and clearance from the circulation. As demonstrated in a seminal paper by the Nobel Prize M.R. Capecchi, even if directly injected in the cell cytoplasm naked NAs do not work at all [2]. Such limitations can be circumvented by gene delivery systems (GDSs) that protect NAs from degradation prolonging their circulation time in the patient’s bloodstream [3]. Over the past decades, several aspects have been investigated and optimized to produce GDSs with clinical utility including (i) scalability and reproducibility of the synthesis process; (ii) high encapsulation of genetic material; (iii) easy monitoring of particle physicochemical characteristics; (iv) internalization processes; (v) and mechanisms of NA release [4,5,6]. Among developed GDSs, cationic lipid/DNA complexes (lipoplexes) have attracted the interest of researchers due to their ability to protect NAs and support intracellular transfection [7]. The conventional method to produce lipoplexes is the bulk mixing process. This method involves a lipid-film hydration followed by extrusion or sonication necessary to generate small unilamellar vesicles, which, subsequently, are incubated with an aqueous buffer containing the NA [8,9]. Thus, the complex formation involves a relatively uncontrolled interaction between liposomes and the genetic material. Currently, this conventional method is still widely used to produce lipoplexes, however the entrapment of the gene materials into liposome by passive-loading yields a low encapsulation efficiency [10]. Moreover, the labor-intensive process and the lack of scalability and reproducibility of these multiple steps represent the major drawbacks of this procedure [11]. Thus, new production strategies to encapsulate NAs need to deal with the issues of reproducibility, scalability of production and encapsulation efficiency [12].

### 1.2. Microfluidic Manufacturing of Lipid Nanoparticles

Recently, lipid nanoparticles (LNPs) (generally composed of cationic lipids for nucleic acid complexation, helper lipids and a PEG-lipid) have emerged as one of the most performing GDSs [13,14,15,16]. LNPs differ from the most conventional lipid-based systems (i.e., lipoplexes) in terms of easy manufacturing process, high scalability, and reproducibility and above all, an advanced nanostructure organization that assures a high encapsulation capacity, elevated biocompatibility and improved transfection efficiency (TE) [17,18]. One factor that highly contributes to the differentiation between the structural properties of lipoplexes and LNPs is the underlying manufacturing process. In-line, microfluidic mixing is emerging as a robust, scalable, and reproducible technique that can be explored to increase the productivity and reproducibility of GDSs [19,20,21,22]. This manufacturing strategy induces rapid mixing of the organic phase containing lipids and the aqueous phase containing NAs, in a controlled environment. This rapid mixing stimulates supersaturation of lipid molecules during the interaction with the NA to generate self-assembled NA-loaded LNPs complexes [23,24]. This production method is considered a bottom-up approach since LNPs self-assemble into the desired structure without the need for further steps, such as size-reduction methods or incubation with payload. The interplay between lipid formulation, packaging properties and microfluidic parameters (such as total flow rate (TFR) or flow rate ratio (FRR)) allows to model the LNPs structure leading, most of the time, to an electron dense morphology that deviates from the traditional multilamellar structure of lipoplexes [25,26]. Currently, data regarding LNPs complexes manufacturing, and structure properties mainly come from the most recent formulation of LNPs encapsulating siRNA and mRNA [27,28,29]. On the other hand, there are only few formulations obtained from the packaging of pDNA [17,30,31]. However, as previously demonstrated for lipoplexes [32,33], optimization of influence factors such as lipid composition, lipid/pDNA weight ratio and microfluidic parameters may dramatically improve the TE of LNPs [34].

### 1.3. Role of Microfluidic Parameters and Effects on Cellular Response

Herein, we explored different factors such as lipid shell composition, amino to phosphate charge ratio, and microfluidic parameters to generate efficient LNP formulations. Given the established greater efficiency of multicomponent lipid-based delivery systems, we generated a novel LNP formulation consisting of two cationic lipids, 1,2-dioleoyl-3-trimethylammonium-propane (DOTAP) and 3β-[N-(N′,N′-dimethylaminoethane)-carbamoyl])-cholesterol (DC-Chol), two helper lipids, cholesterol (Chol) and dioleoylphosphatidylethanolamine (DOPE) and a PEG-lipid, 1,2-dioleoyl-sn-glycero-3-phosphoethanolamine-N-[amino(polyethylene glycol)-2000] (DOPE-PEG2000) at different molar ratio. We first investigated the role of the TFR on the structural features of LNPs. Subsequently, we validated the biological activity of LNPs by testing their TE on Human Embryonic Kidney 293 (HEK-293), aneuploid immortal keratinocytes (HaCaT), epidermoid cervical cancer (CaSki) and immortalized keratinocytes N/TERT cell lines. The results showed that LNPs produced with a lower TFR were generally associated with higher TE and lower cytotoxicity, especially once compared to Lipofectamine™ 3000, the gold standard of lipid transfection [35]. The reported results proved the importance to implement influence factors to enhance the loading of plasmid DNA in LNPs generated through microfluidics. In general, we expected that the proposed approach will be employed for a large-scale production of pDNA-loaded LNPs to get the most of the therapeutic potential of the genetic load, especially in the field of gene therapy and immunotherapy.

## 2. Results and Discussion

**Figure 1:**
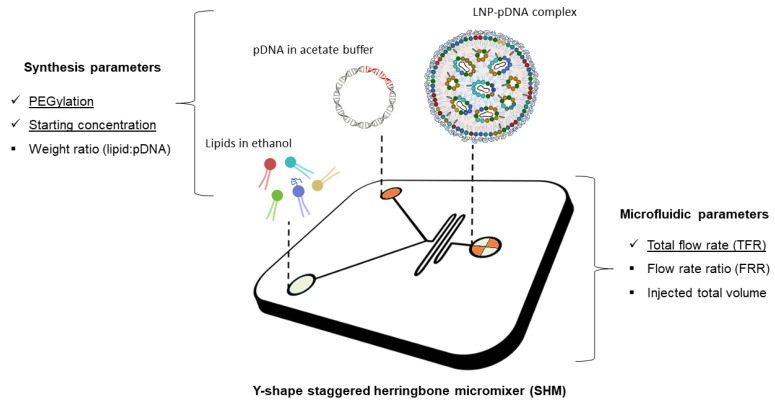
Schematic of lipid nanoparticle-plasmid DNA (LNP-pDNA) complex formulation strategy employing the staggered herringbone micromixer (SHM). Lipids in ethanol are injected in one inlet, while pDNA dissolved in acetate buffer solution is injected in the other inlet. The two solutions meet at the Y-junction of the SHM and undergo a chaotic mixing through the herringbone structure. This phenomenon leads to an increase of lipid solution polarity that generates LNPs encapsulating pDNA. On the sides of the image, two main categories of influential manufacturing factors are reported, i.e., synthesis and microfluidic parameters. Among these, those underlined are the ones that are investigated in the present work.

Previous investigations on lipid-based GDSs showed that multicomponent lipid systems are more efficient and less cytotoxic compared to single-component or binary systems. This was attributed to an unusual ability to escape the endosomal compartment and avoid lysosomal degradation [36,37]. Starting from this assumption, we generated a multicomponent lipid nanoparticle (LNP) system combining two cationic lipids, 1,2-dioleoyl-3-trimethylammonium-propane (DOTAP) and 3β-[N-(N′,N′-dimethylaminoethane)-carbamoyl])-cholesterol (DC-Chol), two helper lipids, cholesterol (Chol) and dioleoylphosphatidylethanolamine (DOPE) and a PEG-lipid, 1,2-dioleoyl-sn-glycero-3-phosphoethanolamine-N-[amino(polyethylene glycol)-2000] (DOPE-PEG2000). Cationic lipids form stable complexes with negatively charged NAs. As for the helper lipids, DOPE has been chosen for the improved packing capabilities conferred by the cone-like geometry of its lipid tails [38]. Furthermore, DOPE with cholesterol also increases the fusogenic properties of lipid vesicles [39]. To generate LNPs, we used a manageable, robust, and highly reproducible technology based on microfluidic mixing through a staggered herringbone micromixer (SHM) (Figure 1).

This method allows to improve the control over the mixing process and shorten the mixing time between the organic lipid solution and the aqueous solution of pDNA. In addition, the structure of the mixer allows an efficient packaging of the two fluids resulting in an expansion of the interface between them [21]. We added the lipid mixture in one inlet of the SHM and the pDNA aqueous solution in the other one. The rapid rise of lipid polarity causes supersaturation and results in fast formation of LNPs. It has been demonstrated that the increase in polarity is governed by the rate of mixing and the ratio of ethanol to aqueous volumes [40]. These two parameters, that are commonly defined as total flow rate (TFR) and flow rate ratio (FRR), can influence size and polydispersity index (PDI) of LNPs.

### 2.1. Physicochemical Characterization of LNPs

**Figure 2:**
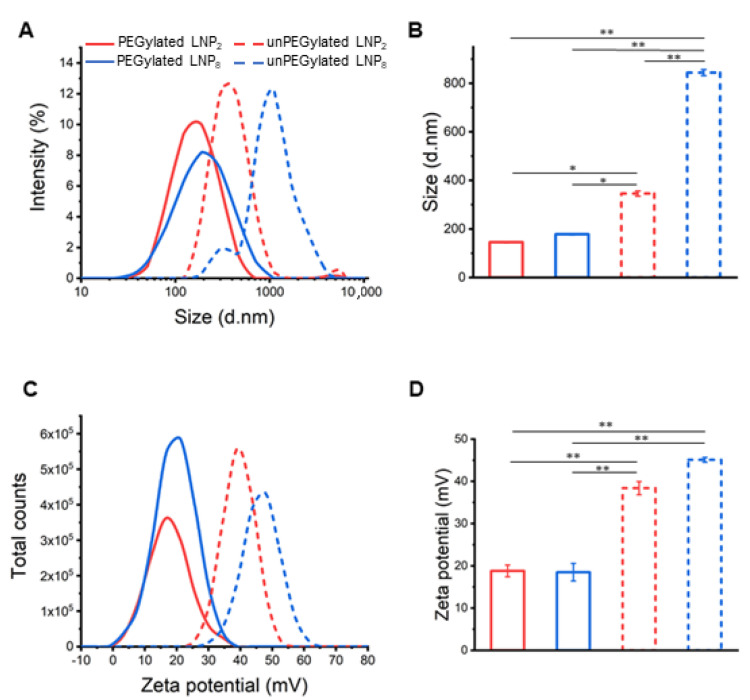
Size (**A**) and zeta potential (**C**) distributions of unPEGylated (dashed lines) and PEGylated (solid lines) LNPs prepared at two different total flow rates (TFR) of 2 mL/min (red lines) and 8 mL/min (blue lines). Average size and zeta potential are displayed in panels (**B**) and (**D**), respectively. PEGylation had a major effect on keeping size of LNPs below the typical threshold size for gene delivery. Moreover, lower TFR produced smaller size and more homogeneous LNPs. Statistical significance was evaluated using Student’s *t*-test: * *p* < 0.05; ** *p* < 0.01.

In this work, we focused on the effect of TFR on the chemical-physical properties and transfection behaviour of LNPs. To this end, we prepared both unPEGylated and PEGylated LNPs at two different TFRs (2 mL/min and 8 mL/min). These formulations shall be identified with subscripts 2 and 8 in the following text (i.e., LNP_2_ and LNP_8_). After the dialysis process for removing the organic solvent, the resulting LNPs were characterized in terms of size, PdI, and zeta-potential through dynamic light scattering (DLS) and transmission electron microscopy (TEM) measurements. As Figure 2 shows, unPEGylated LNP_2_ and LNP_8_ were large, poorly homogeneous in size and highly positively charged.

**Figure 3:**
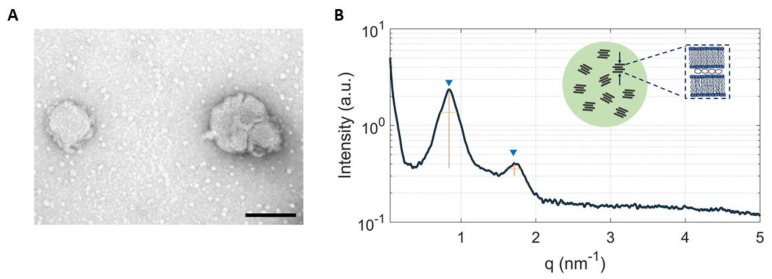
TEM image of PEGylated LNPs (**A**) (scale bar = 100 nm). Synchrotron SAXS pattern of PEGylated LNPs (**B**). The broad peaks arose from the lamellar periodicity along the normal to the lipid bilayer, d, which is the sum of the membrane thickness (dB) and the thickness of the water/DNA layer (dW): d = dB + dW. From the fwhm of the first-ordered Bragg peak we estimated an average domain size made of 4 repeat units. Inset: cartoon describing the nanostructure of LNPs. Randomly oriented lamellar domains coexist within a single particle.

We also observed that TFR had a deep impact on particle size distribution: the higher the TFR, the bigger the size and PdI. As previous studies showed that systems larger in size than 200 nm are incompatible with intravenous administration [41,42] here unPEGylated LNPs were not assessed for their TE. On the other hand, PEGylated LNPs showed a smaller size (145.3 ± 1.6 nm and 178.2 ± 2.7 nm for LNP_2_ and LNP_8_, respectively) and lower zeta potential values (18.5 ± 1.4 and 18.4 ± 2.1). This is in accordance with many studies addressing the aggregation-prevention properties of PEG [43]. In fact, the resulting polymeric layer acts as a physical barrier that limits the interaction between LNPs and prevents aggregation during the manufacturing process [1,30]. Furthermore, even on a smaller length scale, we demonstrated that increasing TFR leads to an increase of particle size and polydispersity, with PdI values that passed from 0.113 ± 0.001 for LNP_2_ to 0.362 ± 0.002 for LNP_8_ (see Table S1 for further details). In parallel, we tested the efficiency of the microfluidic-based synthesis process by performing size and zeta potential measurement on three different batches from the PEGylated LNP_2_ (see Table S2). All three samples showed similar physicochemical properties confirming the high reproducibility of the microfluidic technique. In summary, size, and zeta-potential of PEGylated LNP_2_ and LNP_8_ make them promising candidates for gene delivery applications. Transmission electron microscopy (TEM) measurements have been performed on PEGylated LNPs (Figure 3A). TEM images showed spherically shaped LNPs, validating their uniformity. Synchrotron SAXS was applied to characterize the nanoscale arrangement of PEGylated LNPs (Figure 3B). Two broad Bragg peaks were detected at q_001_ = 0.85 nm^−1^ and q_002_ = 1.70 nm^−1^ (marked by blue arrows in Figure 3).

They arise from the lamellar periodicity along the normal to the lipid bilayer, d, which is the sum of the membrane thickness (d_B_) and the thickness of the water/DNA layer (d_W_): d = d_B_ + d_W_ = 2π/q001 = 7.4 nm. We estimated the average domain size of the layers made of lipids and DNA arrays, Lm, by using the Debye-Scherrer relation Lm = 2π/∆q, where ∆q is the full width at half maximum (fwhm) of the (001) Bragg peak in q space. For the calculation, we used ∆q = [(fwhm)exp^2^ − (fwhm)beam^2^]^1/2^, where (fwhm)exp is the experimental width of the (001) diffraction peak and (fwhm)beam is the width of the intrinsic instrumental resolution function [(fwhm)beam∼6 × 10^−3^ nm^−1^]. We obtained Lm~28 nm that is compatible with an average domain size made of *n* = 4 repeating units. This value is about one order of magnitude lower than that typically found in cationic lipid/DNA complexes (lipoplexes). While a precise characterization of the nanoscale organization of LNPs is beyond the scope of this work and will be the subject of future investigations, our SAXS findings indicate that LNPs are made of multiple short-range domains that are much smaller than the particle size. Such scattering domains are locally ordered along the normal to the lipid bilayer but randomly oriented along one particle radius, thus resembling the structure of crystalline powder samples [44]. Another relevant difference with respect to the SAXS pattern of lipoplexes is the absence of the 1D DNA peak that is a fingerprint of the lipoplex structure and is generated by the one-dimensional in-plane DNA-DNA lattice [45]. This observation suggests that DNA is not densely packed in LNPs and may, therefore, be more easily released intracellularly upon interaction with cellular membranes. In view of biological validation, our next effort was aimed at increasing particle concentration from 0.1 mg/mL up to 0.5 mg/mL. This step would facilitate TE experiments in vitro and would be mandatory in vivo where small volumes (<100 μL) can be administered to mice. To this end, two different approaches were tested. The first strategy consisted in injecting 5× more concentrated lipid and pDNA solutions in the microfluidic chip, while the second one was based on concentrating samples after synthesis by means of centrifugal filters. A comparison of the methods is reported in Figure S1 in the Supplementary Material (SM). As evident, using more concentrated lipids and pDNA increased particle size, probably due to a greater steric hindrance during the mixing process, which compromised the structural organization of the complexes. On the opposite, concentrating samples post-synthesis did not affect particle size distribution and pdI and was therefore used in the following experiments. Both PEGylated LNP_2_ and LNP_8_ showed a final concentration of around 0.5 mg/mL and a similar encapsulation efficiency near 80% (see Table S1 in the SM).

### 2.2. Cell Transfection and Viability

**Figure 4:**
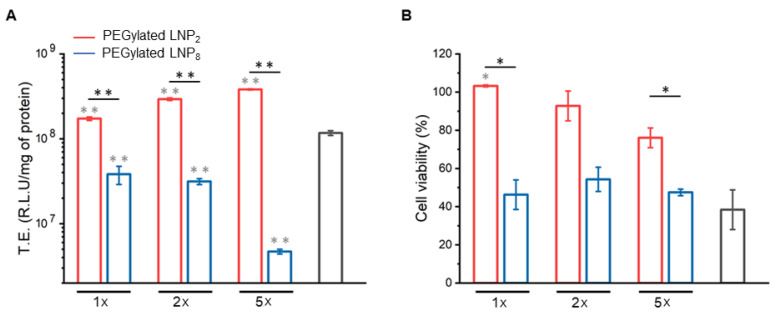
PEGylated LNP_2_ (red histograms) and LNP_8_ (blue histograms) transfection efficiency (TE) (**A**), expressed as Relative Light Unit (R.L.U.) to milligrams of proteins and cell viability (**B**), expressed as percentage with respect of untreated cells, at three different pDNA conditions, namely 1 µg (1×), 2 µg (2×) and 5 µg (5×), towards HEK-293 cells. All values are compared to the positive control LipofectamineTM 3000 (grey histograms) (1×). Statistical significance was evaluated using Student’s *t*-test: * *p* < 0.05; ** *p* < 0.01 (grey asterisk represents significance with respect of Lipofectamine™ 3000, no asterisk means lack of significance).

As a first validation step, we employed HEK-293 cell line to evaluate TE of LNPs by the luciferase reporter technology. This cell line is a suitable model system for TE experiments, especially in cancer research, vaccine development, protein production, and drug testing, since it is able to achieve the post-translational folding and processing needed to generate functional, mature protein from a wide spectrum of nucleic acids [46,47,48,49,50]. In Figure S2, we compared TE and cell viability of HEK-293 cells transfected with LNPs at two different concentrations (i.e., 0.1 mg/mL vs. 0.5 mg/mL). As more concentrated LNPs were more efficient and less cytotoxic than less concentrated ones, they were used in the following experiments. Recently low particle concentration at the cell surface was identified as an overlooked factor in gene delivery [51]. A general conclusion indicated that transfection methods must increase DNA concentration at the target cell surface. We choose three different pDNA concentrations, namely, 1×, 2× and 5× (see Materials and Methods section for further details) to further explore the dose/response effect. Furthermore, as positive control, we used Lipofectamine™ 3000, the gold standard for the transfection of lipid-based systems. However, as LNPs made of are effective only at certain CL–DNA stoichiometric ratios, boosting DNA concentration would result in administration of high doses of cationic lipids to cells, thus potentially affecting cell viability. Therefore, cytotoxicity of PEGylated LNP_2_ and LNP_8_ was also evaluated. Figure 4 compared the TE (panel A) expressed as Relative Light Unit (R.L.U) to milligrams of protein, and cell viability (panel B) of LNP_2_ and LNP_8_.

First, we observed that PEGylated LNP_2_ was roughly one order of magnitude more efficient than LNP_8_ for 1× and 2× pDNA conditions. As for the 5× condition, the gap in TE was much higher, around two orders of difference. In addition, LNP_2_ was significantly more efficient than Lipofectamine™ 3000. TE results also showed that increasing the DNA dose had a variable impact on TE. While TE of PEGylated LNP_2_ increased with increasing of pDNA/well, the opposite trend was found for PEGylated LNP_8_. To better interpret TE results, we investigated the cytotoxicity of LNPs. When HEK293 were treated with PEGylated LNP_2_ cell viability was high and higher than 70% even at the highest DNA dose/well (i.e., 5×). On the other hand, LNP_8_ and Lipofectamine™ 3000 had a major impact on cell viability that was below 50% for each DNA dose. This marked reduction in cell viability may contribute to explain the low TE of PEGylated LNP_8_. In view of the exploitation of LNPs for DNA vaccination and cancer gene therapy, TE and cell viability experiments were replicated with HaCaT and CaSki cell lines (Figure S3 in the SM). HaCaT cells are spontaneously transformed aneuploid immortal keratinocytes derived from adult human skin and can be used as model system of target cells for DNA vaccination purposes [52,53]. On the other hand, CaSki is a human epidermoid cervical cancer cell line frequently used in cancer immunotherapy and vaccination fields. It is noteworthy to observe that both LNP_2_ and LNP_8_ exhibited good TE and low cytotoxicity in both HaCaT and CaSki cells. However, in both cell lines, we found an inverse relationship between TE and DNA dose with respect to HEK-293, i.e., the highest TE was achieved at the lowest DNA dose (1×). Finally, we performed TE and cell viability experiments on immortalized keratinocyte cell line (N/TERT) generated from the transduction of human primary keratinocytes (Figure S4). The treatment with LNP_2_ at 1× DNA condition resulted in lower TE value if compared with Lipofectamine^T^^M^ 3000 but much higher cell viability. This result confirms that certain cell lines (e.g., HEK-293) can be more easily transfected than hard-to-transfect cells and that one transfection reagent does not work well for all the cell lines. On the other hand, LNP formulations described in this study (and in particular LNP_2_) showed a consistent improvement in cell viability in all cell lines tested, compared to Lipofectamine™ 3000.

## 3. Materials and Methods

### 3.1. Microfluidic Preparation of Plasmid Containing LNPs

Cationic lipids 1,2-Dioleoyl-3-trimethyl-ammonium-propane (DOTAP) and (3β-[N-(N′,N′-dimethyl-aminoethane)-carbamoyl])-cholesterol (DC-Chol), zwitterionic lipids dioleoyl phosphatidylethanol-amine (DOPE), Cholesterol and PEG-lipid 1,2-dioleoyl-sn-glycero-3-phosphoethanolamine-N-[amino(polyethylene glycol)-2000, were purchased from Avanti Polar Lipids (Alabaster, AL, USA). pGL3 control plasmid was purchased from Promega (Fitchburg, WI, USA). Lipid nanoparticles (LNPs) were obtained using a Y-shape staggered herringbone micromixer (SHM) (NanoAssemblr^®^ Benchtop from Precision NanoSystems Inc., Vancouver, BC, Canada). Individual lipid stocks were prepared by dissolving cationic lipids DOTAP and DC-Chol, zwitterionic lipid DOPE, cholesterol and a PEG-lipid, DOPE-PEG, in absolute ethanol to have a final total concentration of 12.5 mM. The molar ratio of each lipid was 13, 3:39, 9:31, 9:13, 3:1, 5, respectively, unless otherwise stated. Purified pmirGLO (expressing Firefly luciferase) (Promega, Italy) was dissolved in 25 mM sodium acetate buffer (pH = 4), to 0.2 mg/mL. The syringe pump inserts the two solutions into the microfluidic device, where they interact at the Y-junction. pDNA in aqueous buffer was mixed with lipids in ethanol solution at a flow rate ratio of 3:1 (aqueous to ethanol), at room temperature. Two different total rates (TFR; 2 mL/min and 8 mL/min), were tested to get two LNPs complexes (hereafter flow referred as LNP_2_ and LNP_8_). This process effectively lowered the ethanol concentration to 25% upon leaving the micromixer. The two formulations were produced at the same DNA/lipid weight ratio (Rw = µ0) corresponding to a nitrogen to phosphate charge ratio (N/P; nitrogen from the cationic lipid and phosphate from the nucleic acid) of 3. LNPs complexes were subsequently dialyzed for 19 h against 500 mL of phosphate-buffered saline (PBS) at pH 7.4 with Slide-A-Lyzer. Dialysis cassettes (0.5–3 mL, MWCO 3 kDa, Thermo Scientific, Rockford, MI, USA), to remove the residual ethanol

### 3.2. DLS Characterization of LNPs

Particle size and zeta-potential were measured by dynamic light scattering (DLS) and micro-electrophoresis (ME) at 25 °C using a Zetasizer Nano ZS90 (Malvern, UK). The measurements were made by diluting the sample 1:100 with distilled water and the results were reported as mean ± standard deviation of three independent replicates

### 3.3. Quantification of Plasmid DNA Loading

LNPs encapsulation efficiency (EE%) was measured with the Quant-iT Pico-Green dsDNA assay kit (Thermo Fisher Scientific, Waltham, Massachusetts). LNPs were diluted 300-fold in TE buffer 1× and plated on a Corning^®^ 96 Well Solid Polystyrene Microplate (Sigma-Aldrich, Milan, Italy). The 1% Triton X-100 was added to lyse the LNP and release the encapsulated pDNA. Free pDNA was evaluated by avoiding the LNPs lysis. Quant-iT PicoGreen reagent was inserted to all wells and the samples were incubated at room temperature for 5 min. LNPs fluorescence signal were measured at excitation and emission wavelengths of 475 nm and 500–550 nm, respectively, by using a Glomax Discover System (Promega, Madison, WI, USA). EE% was determined by measuring fluorescence upon addition of PicoGreen to LNPs and comparing it to the value obtained post-lysis, as shown below:

(1) $$\%\;\mathrm{EE}=\frac{\left(Lysed\;LNP-not\;lysed\;LNP\right)}{Lysed\;LNP}\times 100$$

### 3.4. Synchrotron Small Angle X-ray Scattering

Synchrotron small single X-ray scattering (SAXS) measurements were performed at the Austrian SAXS station of the synchrotron light source ELETTRA (Trieste, Italy) by using an automatic sample changer system [54]. Calibration of the detector (Pilatus3 1 M, Dectris, Baden, Switzerland) was carried out by using silver behenate powder (d-spacing = 58.376), q-range was set within 0.05 and 1.5 nm^−1^, exposure times were 10 s (no radiation damage was detected) and temperature was controlled in the vicinity of the capillary. Correction for background, primary beam intensity and detector efficiency were included in the analysis of SAXS patterns.

### 3.5. Transfection Efficiency Assay

Biological evaluation of LNPs obtained was assessed by in vitro transfection in human embryonic kidney 293 (HEK-293) cells (ATCC, Rockville, MD, USA), human immortalized keratinocytes (HaCaT) cells, human cervical cancer (CaSki) cells) (ATCC, Rockville, MD, USA) and immortalized keratinocyte (N/TERT) cell line. Cells were grown in DMEM (HEK-293 and HaCaT) or RPMI (CaSki) supplemented with 10% FBS. In transfection experiments, cells were seeded on 96-well plates (10,000 cells for well). Each treatment has been performed in triplicates. Cell lines were treated for 3 h in Optimem medium (Life Technologies, Carlsbad, CA, USA), with LNP_2_ and LNP_8_ with three different DNA amounts (1×; 2×; 5×), namely 1 µg, 2 µg and 5 µg per three wells (200 mL for well. Lipofectamine™ 3000 was used as positive control at 1× DNA condition following the standardized protocol (Life Technologies, Carlsbad, CA, USA). Then, DMEM 20% FBS was added for HEK-293 and HaCaT cells, and RPMI 20% FBS for CaSKi cells and the cells were incubated at 37 °C. After 48 h, Luciferase expression of cells was measured by means of Luciferase Assay System (Promega, Madison, WI, USA). Briefly, cells were washed in phosphate saline buffer and 20 µL of lysis buffer 1× (Promega) was added in each well. Then, 10 µL of the cell suspension was diluted with 100 µL of luciferase substrate (Promega) and the remaining 10 µL used for BCA assay. The transfection efficiency expressed was determined by Pierce BCA Assay Protein Kit (Thermo Fisher Scientific, Waltham, MA, USA) and expressed as Relative Light Units (RLU) per mg of cell proteins.

### 3.6. Cell Viability Assay

Cell viability of HEK-293, HaCaT and CaSki cells was evaluated by 2, 3-Bis-(2-Methoxy-4-Nitro-5-Sulfophenyl)-2*H*-Tetrazolium-5-Carboxanilide (XTT assay, cell proliferation Kit II, Roche). Cells were seeded on 96-well plates (10,000 cells/well). After 24 h, cells were incubated with LNP_2_ and LNP_8_ complexes in Optimem medium. After 3 h DMEM 20% was added for HEK-293 and HaCaT cells and RPMI 20% for CaSki cells and the cells were incubated for 48 h at 37 °C. Then, 50 µL of XTT solution, previously prepared as indicated in the kit protocol, was added to each well and cells were incubated at 37 °C for 3 h. After that, the absorbance of each well was measured with Glomax Discover System (Promega, Madison, WI, USA), a detection multi-mode instrument with high-performance.

## 4. Conclusions

The development of LNPs encapsulating RNA types has proved that formulations and manufacturing processes need to be adapted to each type of cargo and are not interchangeable. On the other side, preparation of pDNA-loaded LNPs has been only marginally addressed so far. Here, we intended to contribute to fulfil this gap by evaluating the role of influential factors such as concentration of lipids and DNA, PEGylation, and total flow rate. We have demonstrated that these parameters simultaneously affect physicochemical properties and efficiency of pDNA-encapsulating LNPs. This may be relevant in several fields of research ranging from gene therapy to nano-enabled DNA vaccination and cancer immunotherapy.

## Data Availability

The data presented in this study are available from the corresponding authors (D.P. and G.C.) on reasonable request.

## Supplementary Materials

The following are available online at https://www.mdpi.com/article/10.3390/pharmaceutics13081292/s1, Table S1: Chemical and physical characterization of LNP_2_, and LNP_8_. Table S2. Chemical and physical characterization performed on three different batches of PEGylated LNP_2_. Figure S1: Size and zeta potential of LNP_2_ concentrated (i) after synthesis or (ii) derived from 5× concentrated lipid and pDNA solutions. Figure S2: Transfection efficiency and cell viability of HEK-293 cells treated with diluted and concentrated LNP_2_. Figure S3: Transfection efficiency and cell viability of HaCat cells and Caski cells after treatment with LNP_2_, LNP_8_ and Lipofectamine™ 3000. Figure S4: Transfection efficiency and cell viability of N/TERT cells after treatment with LNP_2_ and Lipofectamine™ 3000.

## References

[1] MacLachlan I., Cullis P. (2005). Diffusible-PEG-Lipid Stabilized Plasmid Lipid Particles. Adv. Genet..

[2] Capecchi M.R. (1980). High efficiency transformation by direct microinjection of DNA into cultured mammalian cells. Cell.

[3] Yin H., Kanasty R.L., Eltoukhy A.A., Vegas A.J., Dorkin J.R., Anderson D.G. (2014). Non-viral vectors for gene-based therapy. Nat. Rev. Genet..

[4] Jahn A., Vreeland W.N., Gaitan M., Locascio L.E. (2004). Controlled vesicle self-assembly in microfluidic channels with hydrodynamic focusing. J. Am. Chem. Soc..

[5] Wagner A., Vorauer-Uhl K., Kreismayr G., Katinger H. (2002). The crossflow injection technique: An improvement of the ethanol injection method. J. Liposome Res..

[6] Sanghani A., Kafetzis K., Sato Y., Elboraie S., Fajardo-Sanchez J., Harashima H., Tagalakis A., Yu-Wai-Man C. (2021). Novel PEGylated lipid nanoparticles have a high encapsulation efficiency and effectively deliver MRTF-B siRNA in conjunctival fibroblasts. Pharmaceutics.

[7] Elouahabi A., Ruysschaert J.-M. (2005). Formation and intracellular trafficking of lipoplexes and polyplexes. Mol. Ther..

[8] Digiacomo L., Palchetti S., Pozzi D., Amici A., Caracciolo G., Marchini C. (2018). Cationic lipid/DNA complexes manufactured by microfluidics and bulk self-assembly exhibit different transfection behavior. Biochem. Biophys. Res. Commun..

[9] de la Torre L.G., Pessoa A.C.S.N., de Carvalho B.G., Taketa T.B., Eş I., Perli G. (2021). Bulk and Microfluidic Synthesis of Stealth and Cationic Liposomes for Gene Delivery Applications. DNA Vaccines.

[10] Grimaldi N., Andrade F., Segovia N., Ferrer-Tasies L., Sala S., Veciana J., Ventosa N. (2016). Lipid-based nanovesicles for nanomedicine. Chem. Soc. Rev..

[11] MacLachlan I. (2007). Liposomal formulations for nucleic acid delivery. Antisense drug technology: Principles, strategies, and applications. Antisense Drug Technol..

[12] Evers M.J.W., Kulkarni J., Van Der Meel R., Cullis P.R., Vader P., Schiffelers R.M. (2018). State-of-the-Art Design and Rapid-Mixing Production Techniques of Lipid Nanoparticles for Nucleic Acid Delivery. Small Methods.

[13] Kimura N., Maeki M., Sato Y., Ishida A., Tani H., Harashima H., Tokeshi M. (2020). Development of a Microfluidic-Based Post-Treatment Process for Size-Controlled Lipid Nanoparticles and Application to siRNA Delivery. ACS Appl. Mater. Interfaces.

[14] Kauffman K.J., Dorkin J.R., Yang J.H., Heartlein M.W., DeRosa F., Mir F.F., Fenton O.S., Anderson D.G. (2015). Optimization of lipid nanoparticle formulations for mRNA delivery in vivo with fractional factorial and definitive screening designs. Nano Lett..

[15] Pozzi D., Marchini C., Cardarelli F., Rossetta A., Colapicchioni V., Amici A., Montani M., Motta S., Brocca P., Cantù L. (2013). Mechanistic understanding of gene delivery mediated by highly efficient multicomponent envelope-type nanoparticle systems. Mol. Pharm..

[16] Samaridou E., Heyes J., Lutwyche P. (2020). Lipid nanoparticles for nucleic acid delivery: Current perspectives. Adv. Drug Deliv. Rev..

[17] Kulkarni J., Myhre J.L., Chen S., Tam Y.Y.C., Danescu A., Richman J., Cullis P.R. (2017). Design of lipid nanoparticles for in vitro and in vivo delivery of plasmid DNA. Nanomed. Nanotechnol. Biol. Med..

[18] Eygeris Y., Patel S., Jozic A., Sahay G. (2020). Deconvoluting lipid nanoparticle structure for messenger RNA delivery. Nano Lett..

[19] Di Santo R., Digiacomo L., Palchetti S., Palmieri V., Perini G., Pozzi D., Papi M., Caracciolo G. (2019). Microfluidic manufacturing of surface-functionalized graphene oxide nanoflakes for gene delivery. Nanoscale.

[20] Di Santo R., Quagliarini E., Palchetti S., Pozzi D., Palmieri V., Perini G., Papi M., Capriotti A.L., Laganà A., Caracciolo G. (2019). Microfluidic-generated lipid-graphene oxide nanoparticles for gene delivery. Appl. Phys. Lett..

[21] Belliveau N., Huft J., Lin P.J., Chen S., Leung A.K., Leaver T.J., Wild A.W., Lee J.B., Taylor R.J., Tam Y.K. (2012). Microfluidic synthesis of highly potent limit-size lipid nanoparticles for in vivo delivery of siRNA. Mol. Ther. Nucleic Acids.

[22] Zhigaltsev I.V., Belliveau N., Hafez I., Leung A.K.K., Huft J., Hansen C., Cullis P.R. (2012). Bottom-up design and synthesis of limit size lipid nanoparticle systems with aqueous and triglyceride cores using millisecond microfluidic mixing. Langmuir.

[23] Leung A.K.K., Tam Y.Y.C., Chen S., Hafez I.M., Cullis P.R. (2015). Microfluidic mixing: A general method for encapsulating macromolecules in lipid nanoparticle systems. J. Phys. Chem. B.

[24] Chen D., Love K.T., Chen Y., Eltoukhy A.A., Kastrup C., Sahay G., Jeon A., Dong Y., Whitehead K.A., Anderson D.G. (2012). Rapid discovery of potent siRNA-containing lipid nanoparticles enabled by controlled microfluidic formulation. J. Am. Chem. Soc..

[25] Maeki M., Fujishima Y., Sato Y., Yasui T., Kaji N., Ishida A., Tani H., Baba Y., Harashima H., Tokeshi M. (2017). Understanding the formation mechanism of lipid nanoparticles in microfluidic devices with chaotic micromixers. PLoS ONE.

[26] Roces C.B., Lou G., Jain N., Abraham S., Thomas A., Halbert G.W., Perrie Y. (2020). Manufacturing considerations for the development of lipid nanoparticles using microfluidics. Pharmaceutics.

[27] Kulkarni J.A., Darjuan M.M., Mercer J.E., Chen S., van der Meel R., Thewalt J.L., Tam Y.Y.C., Cullis P.R. (2018). On the formation and morphology of lipid nanoparticles containing ionizable cationic lipids and siRNA. ACS Nano.

[28] Leung A.K.K., Hafez I.M., Baoukina S., Belliveau N., Zhigaltsev I.V., Afshinmanesh E., Tieleman D.P., Hansen C.L., Hope M.J., Cullis P.R. (2012). Lipid nanoparticles containing siRNA synthesized by microfluidic mixing exhibit an electron-dense nanostructured core. J. Phys. Chem. C.

[29] Walsh C., Ou K., Belliveau N.M., Leaver T.J., Wild A.W., Huft J., Lin P.J., Chen S., Leung A.K., Lee J.B. (2014). Microfluidic-Based Manufacture of siRNA-Lipid Nanoparticles for Therapeutic Applications, in Drug Delivery System.

[30] Zukancic D., Suys E.J.A., Pilkington E.H., Algarni A., Al-Wassiti H., Truong N.P. (2020). The importance of poly(Ethylene glycol) and lipid structure in targeted gene delivery to lymph nodes by lipid nanoparticles. Pharmaceutics.

[31] Kimura S., Khalil I.A., Elewa Y.H., Harashima H. (2021). Novel lipid combination for delivery of plasmid DNA to immune cells in the spleen. J. Control. Release.

[32] Marchini C., Pozzi D., Montani M., Alfonsi C., Amici A., Amenitsch H., De Sanctis S.C., Caracciolo G. (2010). Tailoring lipoplex composition to the lipid composition of plasma membrane: A Trojan horse for cell entry?. Langmuir.

[33] Caracciolo G., Pozzi D., Caminiti R., Marchini C., Montani M., Amici A., Amenitsch H. (2008). Enhanced transfection efficiency of multicomponent lipoplexes in the regime of optimal membrane charge density. J. Phys. Chem. B.

[34] Shepherd S.J., Issadore D., Mitchell M.J. (2021). Microfluidic formulation of nanoparticles for biomedical applications. Biomaterials.

[35] Cardarelli F., Digiacomo L., Marchini C., Amici A., Salomone F., Fiume G., Rossetta A., Gratton E., Pozzi D., Caracciolo G. (2016). The intracellular trafficking mechanism of Lipofectamine-based transfection reagents and its implication for gene delivery. Sci. Rep..

[36] Caracciolo G., Pozzi D., Caminiti R., Marchini C., Montani M., Amici A., Amenitsch H. (2006). DNA release from cationic liposome/DNA complexes by anionic lipids. Appl. Phys. Lett..

[37] Caracciolo G., Caminiti R., Digman M.A., Gratton E., Sanchez S. (2009). Efficient escape from endosomes determines the superior efficiency of multicomponent lipoplexes. J. Phys. Chem. B.

[38] Caracciolo G., Amenitsch H. (2012). Cationic liposome/DNA complexes: From structure to interactions with cellular membranes. Eur. Biophys. J..

[39] Pan J., Heberle F., Tristram-Nagle S., Szymanski M., Koepfinger M., Katsaras J., Kucerka N. (2012). Molecular structures of fluid phase phosphatidylglycerol bilayers as determined by small angle neutron and X-ray scattering. Biochim. Biophys. Acta (BBA) Biomembr..

[40] Song Y., Hormes J., Kumar C.S.S.R. (2008). Microfluidic Synthesis of Nanomaterials. Small.

[41] Prabha S., Zhou W.-Z., Panyam J., Labhasetwar V. (2002). Size-dependency of nanoparticle-mediated gene transfection: Studies with fractionated nanoparticles. Int. J. Pharm..

[42] Harashima H., Hiraiwa T., Ochi Y., Kiwada H. (1995). Size Dependent Liposome Degradation in Blood: In vivo/In vitro Correlation by Kinetic Modeling. J. Drug Target..

[43] Milton H.J., Martin N.E., Modi M. (2001). PEGylation: A novel process for modifying pharmacokinetics. Clin. Pharm..

[44] Rapaport H., Kuzmenko I., Lafont S., Kjaer K., Howes P.B., Als-Nielsen J., Lahav M., Leiserowitz L. (2001). Cholesterol Monohydrate Nucleation in Ultrathin Films on Water. Biophys. J..

[45] Pozzi D., Caracciolo G., Caminiti R., De Sanctis S.C., Amenitsch H., Marchini C., Montani M., Amici A. (2009). Toward the Rational Design of Lipid Gene Vectors: Shape Coupling between Lipoplex and Anionic Cellular Lipids Controls the Phase Evolution of Lipoplexes and the Efficiency of DNA Release. ACS Appl. Mater. Interfaces.

[46] Stepanenko A., Dmitrenko V. (2015). HEK293 in cell biology and cancer research: Phenotype, karyotype, tumorigenicity, and stress-induced genome-phenotype evolution. Gene.

[47] Hudecova S., Lencesova L., Csaderova L., Sirova M., Cholujova D., Cagala M. (2011). Chemically mimicked hypoxia modulates gene expression and protein levels of the sodium calcium exchanger in HEK 293 cell line via HIF-1α. Gen. Physiol. Biophys..

[48] Le Ru A., Jacob D., Transfiguracion J., Ansorge S., Henry O., Kamen A.A. (2010). Scalable production of influenza virus in HEK-293 cells for efficient vaccine manufacturing. Vaccine.

[49] Liu X., Shan K., Shao X., Shi X., He Y., Liu Z., Jacob J.A., Deng L. (2021). Nanotoxic Effects of Silver Nanoparticles on Normal HEK-293 Cells in Comparison to Cancerous HeLa Cell Line. Int. J. Nanomed..

[50] Thomas P., Smart T.G. (2005). HEK293 cell line: A vehicle for the expression of recombinant proteins. J. Pharmacol. Toxicol. Methods.

[51] Palchetti S., Pozzi D., Marchini C., Amici A., Andreani C., Bartolacci C., Digiacomo L., Gambini V., Cardarelli F., Di Rienzo C. (2017). Manipulation of lipoplex concentration at the cell surface boosts transfection efficiency in hard-to-transfect cells. Nanomed. Nanotechnol. Biol. Med..

[52] Boukamp P., Petrussevska R.T., Breitkreutz D., Hornung J., Markham A., Fusenig N.E. (1988). Normal keratinization in a spontaneously immortalized aneuploid human keratinocyte cell line. J. Cell Biol..

[53] Carter T.H., Liu K., Ralph W., Chen D., Qi M., Fan S., Yuan F., Rosen E.M., Auborn K.J. (2002). Diindolylmethane Alters Gene Expression in Human Keratinocytes In Vitro. J. Nutr..

[54] Haider R., Sartori B., Radeticchio A., Wolf M., Dal Zilio S., Marmiroli B., Amenitsch H. (2021). µDrop: A system for high-throughput small-angle X-ray scattering measurements of microlitre samples. J. Appl. Crystallogr..

