# Supplementary material for: Microfluidic Formulation of DNA-Loaded Multicomponent Lipid Nanoparticles for Gene Delivery
Source: Pharmaceutics. 2021 Aug 19;13(8):1292. doi: 10.3390/pharmaceutics13081292 (PMC8400491; doi:10.3390/pharmaceutics13081292)
Supplement: Supplementary file 1 [file pharmaceutics-13-01292-s001.zip › pharmaceutics-1309793-supplementary.pdf]

pharmaceutics

Supplementary Materials: Microfluidic Formulation of DNA-Loaded Multicomponent Lipid Nanoparticles for Gene Delivery

Erica Quagliarini, Serena Renzi, Luca Digiacomo, Francesca Giulimondi, Barbara Sartori, Heinz Amenitsch, Valentina Tassinari, Laura Masuelli, Roberto Bei, Lishan Cui, Junbiao Wang, Augusto Amici, Cristina Marchini, Daniela Pozzi and Giulio Caracciolo

Table S1. Chemical and physical characterization of LNP_2, and LNP_8.

	**LNP_2**	**LNP_8**
Size (d.nm)	145.3 ± 1.6	178.2 ± 2.7
Zeta potential (mV)	18.5 ± 1.4	18.4 ± 2.1
PdI	0.113 ± 0.001	0.362 ± 0.002
Encapsulation Effeiciency	76.8	72.7

Table S2. Chemical and physical characterization performed on three different batches of PEGylated LNP_2,.

	**Batch 1**	**Batch 2**	**Batch 3**
Size (d.nm)	145.3 ± 1.6	137.5 ± 0.67	145.6 ± 1.78
Zeta potential (mV)	18.5 ± 1.4	21.1 ± 2.4	14.5 ± 3.4
PdI	0.113 ± 0.001	0.238 ± 0.002	0.225 ± 0.002

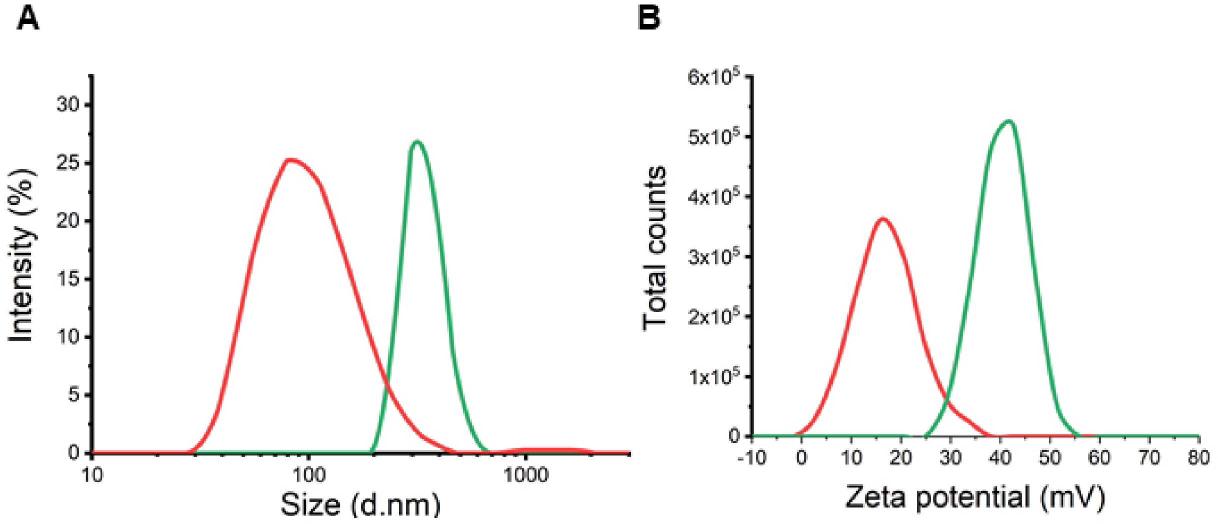

Figure S1. Size (panel **A**) and zeta potential (panel **B**) of LNP_2 concentrated 5× after synthesis (red lines) and LNP_2 derived from 5× concentrated lipid and pDNA solutions (green lines).

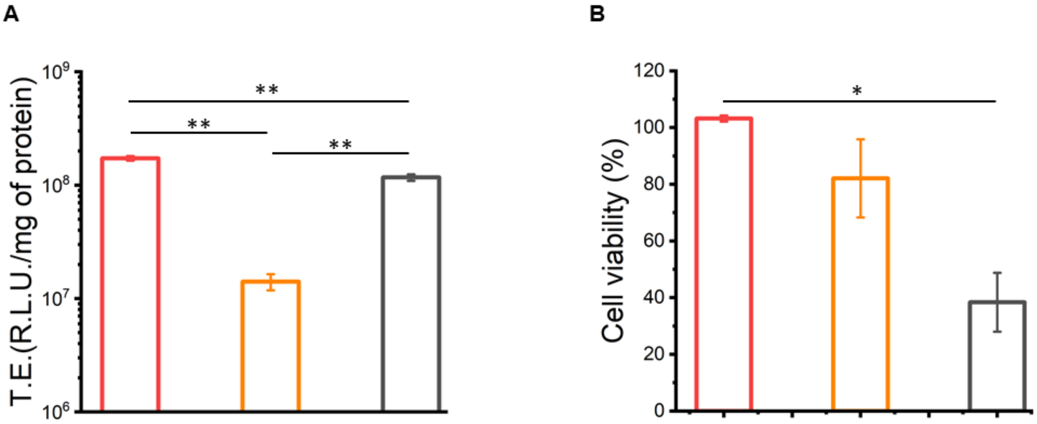

Figure S2. Transfection efficiency (T.E.) expressed as Relative Light Unit (R.L.U) to milligrams of proteins (**A**) and cell viability, expressed as percentage with respect to untreated cells, of LNP_2 at 0.5 mg/mL (red histograms) and LNP_2 at 0.1 mg/mL (orange histograms), towards HEK-293 cells. All values are compared to Lipofectamine (grey histograms). Statistical significance was evaluated using Student's t-test: *$p < 0.05$; **$p < 0.01$ (no asterisk means lack of significance).

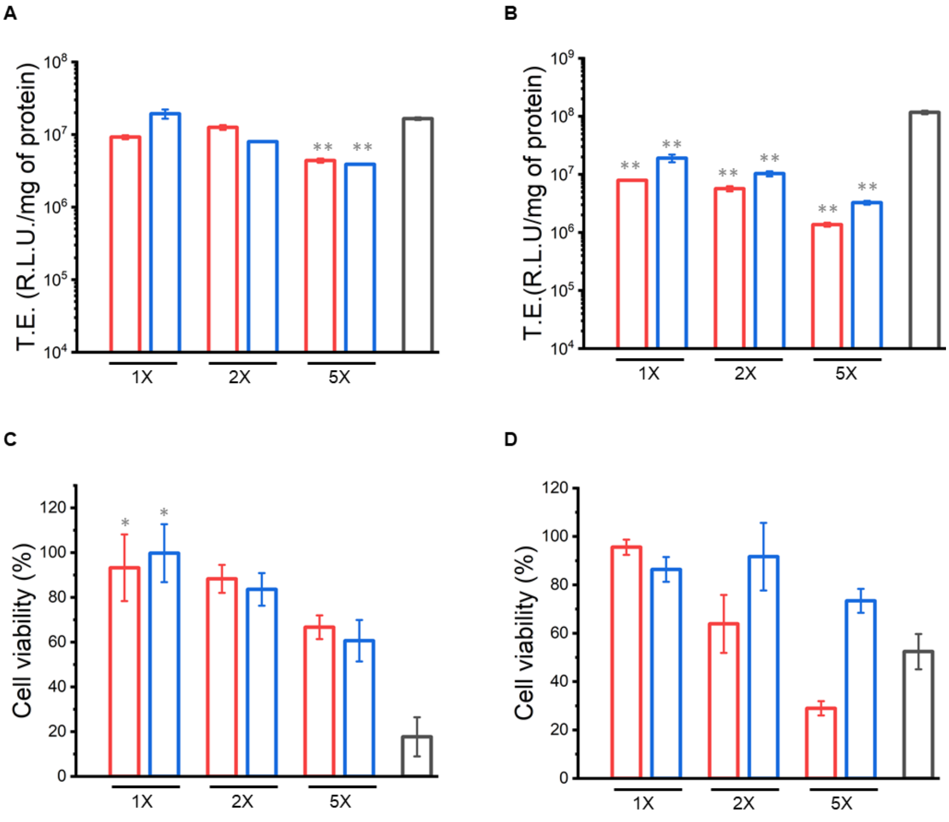

Figure S3. LNP_2 (red histograms) and LNP_8 (blue histograms) transfection efficiency (TE) expressed as Relative Light Unit (R.L.U.) to milligrams of proteins, towards HaCaT cells (**A**) and CaSki cells (**B**) compared to T.E. of Lipofectamine™ 3000 (grey histograms). Cell viability of HaCaT cells (**C**) and CaSki cells (**D**) after treatment with LNP_2, LNP_8 and Lipofectamine™ 3000, expressed as percentage with respect of untreated cells. Statistical significance was evaluated using Student's t-test: *$p < 0.05$; **$p < 0.01$ (grey asterisk represents significance with respect of Lipofectamine ™ 3000, no asterisk means lack of significance).

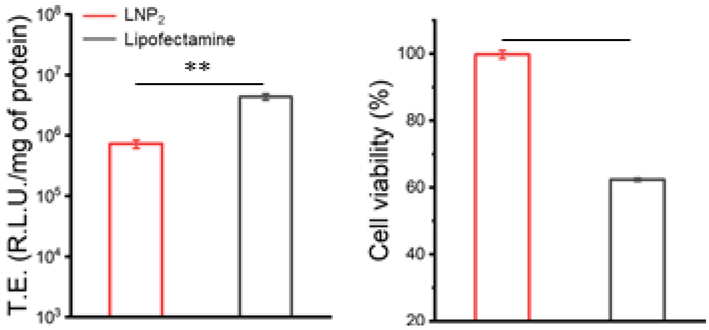

Figure S4. LNP2 (red histograms) and Lipofectamine™ 3000 (grey hystograms) transfection efficiency (TE) expressed as Relative Light Unit (R.L.U.) to milligrams of proteins, towards N/TERT cells (**A**). Cell viability of N/TERT cells (**B**) after treatment with LNP2 and Lipofectamine™ 3000, expressed as percentage with respect of untreated cells. Statistical significance was evaluated using Student's t-test: $^{**}p < 0.01$ (no asterisk means lack of significance).
